# Whole-Genome Phylogenetic Characterization of Human Parainfluenza Virus Type 4 Circulating in St. Petersburg, Russia

**DOI:** 10.3390/v18050497

**Published:** 2026-04-24

**Authors:** Oula Mansour, Artem V. Fadeev, Alexander A. Perederiy, Andrey D. Ksenafontov, Anastasiia Y. Boyarintseva, Daria M. Danilenko, Dmitry A. Lioznov, Andrey B. Komissarov

**Affiliations:** 1 Smorodintsev Research Institute of Influenza, 197376 Saint Petersburg, Russia; olamansor1995@gmail.com (O.M.); afadeew@gmail.com (A.V.F.); gilagalex@gmail.com (A.A.P.); ksenandrey@yandex.ru (A.D.K.); nastb08@gmail.com (A.Y.B.); daria.baibus@gmail.com (D.M.D.); dlioznov@yandex.ru (D.A.L.); 2 Department of Infectious Diseases and Epidemiology, Pavlov First Saint Petersburg State Medical University, 197022 Saint Petersburg, Russia

**Keywords:** human parainfluenza virus 4a, human parainfluenza virus 4b, severe acute respiratory infection, hemagglutinin-neuraminidase, genetic p-distance, phylogenetic analysis

## Abstract

Human parainfluenza virus type 4 (hPIV4) remains poorly characterized compared with other hPIV serotypes and information on its genomic diversity is particularly limited for Russia and Eastern Europe. In this study, we report the first complete genome sequences of hPIV4 isolates from Russia and place them in the context of global hPIV4 genetic diversity. Eight hPIV4 viruses were isolated in cell culture from respiratory samples collected from hospitalized children in Saint Petersburg between 2017/2018 and 2023/2024. Complete viral genomes were recovered using a metagenomic whole-genome amplification approach based on SMART-9N technology. Phylogenetic analysis of 178 complete hPIV4 genomes showed clear separation into hPIV4a (*n* = 132) and hPIV4b (*n* = 46) subtypes. Based on genetic distance approach, hPIV4a formed two major clusters, with the dominant cluster B subdivided into four subclusters (B1–B4); and subcluster B4 further resolved into four genetic lineages. All Russian isolates belonged to the subcluster B4 and were distributed among multiple co-circulating lineages. In contrast, hPIV4b genomes segregated into three distinct clusters, reflecting structured genetic diversity within the subtype. Collectively, this study provides, to the best of our knowledge, the first p-distance-based framework for hPIV4 whole-genome classification and contributes new complete genome sequences for an underrepresented region.

## 1. Introduction

Human parainfluenza viruses (hPIVs) are a group of spherical, enveloped, single negative-sense stranded RNA viruses, which belong to the family *Paramyxoviridae* [1]. According to the genetic and serological characteristics, they can be divided into four serotypes, among which hPIV1 and hPIV3 are classified into the genus Respirovirus, whereas hPIV2 and hPIV4 are classified into the genus Orthorubulavirus, with hPIV4 designated as Orthorubulavirus hominis according to the current ICTV taxonomy [2,3].

The clinical manifestations associated with hPIV vary from mild upper respiratory illnesses to severe pneumonia [4,5]. Seasonal hPIV epidemics result in a significant burden of disease in children and have been estimated to account for 40% of childhood hospitalizations for lower respiratory tract illnesses (LRTIs) and 75% of croup cases [6]. However, each serotype has distinct clinical and epidemiological manifestations. hPIV1 and hPIV2 are generally known to cause croup, whereas lower respiratory tract infections, such as bronchiolitis and pneumonia, are frequently observed in infants with hPIV3 infection [7,8]. hPIV4 has been considered less clinically important as it has been mostly associated with mild illnesses [9,10,11,12]. The seasonality of each hPIV serotype shows geographic variation. However, hPIV1 and hPIV2 infections are more commonly detected in the fall season, hPIV3 infections usually occur in spring and early summer, while the seasonal patterns of hPIV4 are not well described [13,14,15,16].

hPIV4 was first isolated and identified in 1959 by Johnson et al., from a male college student with a mild upper respiratory tract infection [17]. hPIV4 is subdivided into two antigenic subtypes, hPIV4a and hPIV4b, based on hemagglutination inhibition and monoclonal antibody reactivity [18], although hPIV4a dominated [19].

The genomes of hPIV4 subtypes 4a and 4b exceed 17 kb in length. Their viral genomes encode for nucleocapsid (NP), phospho (P), non-structural (V), matrix (M), fusion (F), hemagglutinin–neuraminidase (HN), and large (L) proteins [1]. Like other *Paramyxoviridae* members, it possesses two key surface glycoproteins: the fusion (F) protein and the hemagglutinin–neuraminidase (HN) protein, which are recognized as the primary antigens. These glycoproteins are crucial for the virus’s ability to cause disease and establish infection. The F protein facilitates the fusion of the viral envelope with the host cell membrane, enabling entry into the cell, while the HN protein serves as the attachment factor, binding to cellular receptors such as sialic acid residues to mediate viral attachment [2,20].

On the contrary to the other three serotypes, hPIV4 remains poorly characterized and received comparatively limited research attention, primarily due to (i) its apparently low prevalence rate compared to other hPIVs [21], (ii) it is associated with asymptomatic or mild respiratory tract disease [22], (iii) low recovery in cell culture attributed to difficulties in propagation and the lack of cytopathic effects in most cell lines [12,23]. It is also known to exhibit weak or inconsistent hemagglutination activity, which may complicate its laboratory detection and characterization [12,18]. However, recent studies have reported that it can cause lower respiratory tract infections, such as pneumonia and bronchiolitis, in children and elderly [24,25,26] and in immunocompetent individuals [27,28]. Additionally, outbreaks in limited geographical areas caused by hPIV4 were described, as well [29,30]. Moreover, recent epidemiological studies have shown that the clinical presentations of hPIV4 are similar to those observed with other hPIV serotypes [25,31]. Collectively, these studies have suggested that both the prevalence and clinical characteristics of hPIV4 as a cause of respiratory illness may have been underestimated and remains incompletely characterized.

Despite the growing awareness of hPIV4 as a potential cause of respiratory illness, information on its genetic diversity and molecular characteristics is still limited, particularly for strains circulating in Russia and Eastern Europe. In this study, we report the first complete genome sequences of hPIV4 isolates from Russia, obtained from cell culture propagated viruses. Using a metagenomic whole-genome amplification approach based on the SMART (Switching Mechanism at the 5′ end of RNA Template) technology [32], which has not previously been applied to hPIV sequencing, we aimed to analyze the genetic diversity, phylogenetic relationships, and molecular features of locally circulating hPIV4 strains in comparison with global references.

## 2. Materials and Methods

### 2.1. Ethics Statement

The samples utilized in this study were collected as part of an approved ongoing hospital surveillance program conducted by the Smorodintsev Research Institute of Influenza. The Global Influenza Hospital Surveillance Network (GIHSN) study [33] received approval from the Smorodintsev Research Institute of Influenza Local Ethics Committee (protocol No. 152, 18 June 2020). The study was conducted in accordance with the principles of the Declaration of Helsinki [34]. Written informed consent was obtained from all participants in compliance with the Ministry of Health of the Russian Federation’s order dated 21 July 2015 (#474n).

### 2.2. Sample Collection

Clinical samples including oropharyngeal and nasopharyngeal swabs were collected from hospitalized children aged from 1 month to 5 years with respiratory tract infections, admitted as part of project “Complex approach to genetic characterization and early identifications of pathogens with epidemic and pandemic potential using metagenomic sequencing” during the epidemic seasons from 2017/18 to 2023/24. The samples were received from three hospitals: Clinical Infectious Disease Hospital named after S.P. Botkin, City Children’s Hospital of St. Olga, and St. Petersburg State City Children’s Clinical Hospital No. 5 named after N.F. Filatov, Saint Petersburg, Russia. The samples were collected in virus transport medium (UTM-RT, Copan, Murrieta, CA, USA) and transferred to the Smorodintsev Research Institute of Influenza, Saint Petersburg, under cold-chain conditions for further analysis. Specimens were registered and stored at −70 °C pending molecular analysis. All procedures were performed in accordance with the relevant guidelines and regulations.

### 2.3. Viruses and Cells

The cell lines utilized in this study are LLC-MK2 (rhesus monkey kidney epithelial cells), obtained from the Federal State Budgetary Scientific Institute of Experimental Medicine (St. Petersburg, Russia) and MA-104 (African green monkey kidney cells), obtained from the Russian Type Culture Collection (RTCC, St. Petersburg, Russia). Human parainfluenza virus 4a (strain M-25) and 4b (strain CH 19503) from the American Type Culture Collection (ATCC, Manassas, VA, USA), were obtained through the International Reagent Resource, Influenza Division, WHO Collaborating Center for Surveillance, Epidemiology and Control of Influenza, Centers for Disease Control and Prevention (Atlanta, GA, USA) and used as positive controls.

### 2.4. Virus Isolation

A total of 100 μL of the viral supernatant transport medium was propagated on each of LLC-MK2 and MA-104 cell lines in 24-well tissue culture plates. After 1 h of viral absorption at 37 °C, maintenance media of both Dulbecco’s modified Eagle’s medium (DMEM, with L-glutamine, glucose 1 g/L; BioloT Ltd., St. Petersburg, Russia) and Minimum Essential Medium α (α-MEM, with L-glutamine; BioloT Ltd., St. Petersburg, Russia) containing 2 µg/mL of TPCK-treated trypsin (Sigma-Aldrich, St. Louis, MO, USA) and albumin were added onto LLC-MK2 and MA-104 plates, respectively. The plates were incubated at 37 °C, 5% CO_2_ incubator for 21 days. Every 48 h, the dynamics of CPE development were monitored. Hemagglutination test with 0.5% guinea pig, chicken, and human erythrocytes was performed at the end of the incubation period. When specific cytopathic effect (CPE) was observed in both cell lines, the supernatants were harvested and viral isolation was confirmed by RT-PCR analysis.

### 2.5. RNA Extraction

Total RNA was extracted from aliquots of cell suspensions collected from culture infected with the hPIV4, using the RNA extraction Kit MagnaPrime FAST-R (NextBio^®^, Moscow, Russia) on AutoPure-96 automatic station (Allsheng, Hangzhou, China), according to the manufacturer’s recommendations. RNA pellets were eluted in 100 μL of elution buffer. Viral RNA was tested by real-time PCR using commercial kits “AmpliSens^®^ ARVI-screen-FL” assay (Central Research Institute of Epidemiology, Moscow, Russia), in accordance with the manufacturer’s instructions.

### 2.6. Whole-Genome Amplification Based on the SMART-9N Technology

The SMART-9N amplification method is a modification of the SMART (Switching Mechanism at the 5′ End of RNA Template) cDNA synthesis approach [35], in which the “9N” refers to a primer containing nine random nucleotides, initiating reverse transcription and enabling unbiased capture of random RNA molecules. The complete hPIV4 genomes were amplified using this technique. Briefly, first-strand cDNA synthesis was performed using M-MLV reverse transcriptase from the Magnus Reverse Transcriptase kit (Evrogen, Moscow, Russia), according to the manufacturer’s instructions with a 9N-containing primer (5′-AAGCAGTGGTATCAACGCAGAGTNNNNNNNNN-3′) and a template-switching oligonucleotide (TSO) (5′-AAGCAGTGGTATCAACGCAGAGTACrGrGrG-3′), which adds a defined adapter sequence to the 5′ ends of cDNA during reverse transcription. This enables recovery of full-length viral cDNA from the 3′ or terminal regions of RNA templates through the enzyme’s natural template-switching activity. Reverse transcription protocol was as follows: 25 °C for 10 min, 42 °C for 3 h, 85 °C for 5 min. Following cDNA synthesis, second-strand synthesis and PCR amplification were performed using the BioMaster LR HS-PCR (2X) kit (Biolabmix, Novosibirsk, Russia) and the SMART primer (5′-AAGCAGTGGTATCAACGCAGAGT-3′). The amplification protocol was as follows: 25 °C for 2 min, 95 °C for 5 min, followed by 49 cycles of 94 °C 15 s, 62 °C 15 s and 68 °C 4 min 30 s; followed by 68 °C 15 min, melt curve 75 °C to 90 °C.

### 2.7. Sequencing of Amplicons

Amplicons from PCR reactions were subsequently subjected to library preparation. DNA libraries were prepared using the MGIEasy Fast PCR-free FS DNA Library Prep Set (MGI Tech, Shenzhen, China) according to the manufacturer’s instructions, with specific modifications to optimize fragment size for single-end sequencing. These included extended fragmentation time (25 min vs. 7.5 min in the original protocol), adjustment of bead-to-DNA ratio during cleanup (1.2× vs. 0.8×), and prolonged adapter ligation time (17 min vs. 10 min). These modifications were introduced to obtain shorter DNA libraries suitable for SE100 sequencing (target library size ~300 bp, including 100–150 bp insert length). Whole-genome sequencing was performed on the MGI DNBSEQ G-400 platform (MGI Tech, Shenzhen, China) in single-end read mode with a read length of 100 bp.

### 2.8. Sequence Assembly and Analysis

The BWA v. 0.7.17, Samtools v. 1.19.2, and Ivar v.1.4.2 were used to assemble the consensus nucleotide sequences obtained for each of the analyzed samples along with custom Python v. 3.12 scripts. Genome sequencing was considered successful if ≥95% of genome length was covered and median sequencing depth exceeded ≥10×. Multiple sequence alignment of the complete genomes was generated using MAFFT software with default parameters [36], implemented in UGENE v53. Phylogenetic analysis of whole-genome sequences was performed using the maximum-likelihood (ML, GTR + G) method implemented in IQ-TREE v3.0.1. Branch support was estimated by 1000 bootstrap replicates. The generated phylogenetic trees were visualized with FigTree v1.4.4 (http://tree.bio.ed.ac.uk/software/figtree, accessed on 10 March 2026). To assess the degree of divergence, mean pairwise genetic distances were calculated using MEGA v11.0.13 [37]. Genetic distance thresholds were adopted from Mao et al. (2012) and defined as follows: mean p-distance ≥ 0.045 was used to delineate major clusters, distances between 0.019 and 0.045 were considered indicative for subclusters, distances between 0.010 and 0.019 define distinct genetic lineages and distances ≤ 0.010 were interpreted as sequences belonging to the same lineage [38].

In addition to whole-genome phylogenetic analysis, subtype-specific maximum-likelihood phylogenetic trees based on the complete HN gene were constructed for hPIV4a and hPIV4b using RAxML (GTR + G substitution model) in combination with TreeSub (https://github.com/tamuri/treesub, accessed on 10 March 2026). This integrated approach applies ancestral state reconstruction (ASR) via PAML to infer amino acid substitutions along individual branches, enabling identification of cluster-, subcluster- and genetic lineage-defining amino acid changes in the HN protein.

Potential N-glycosylated sites (Asn-X-Ser/Thr) in HN protein were predicted using the NetNGlyc server (https://services.healthtech.dtu.dk/service.php?NetNGlyc-1.0, accessed on 10 March 2026). Sites with threshold scores above 0.5 were characterized glycosylated.

### 2.9. Strains in the Present Study

To conduct the phylogenetic analysis of the hPIV4 genome, reference strains of hPIV4a and hPIV4b were downloaded from GenBank [https://www.ncbi.nlm.nih.gov/genbank/; last accessed on 1 March 2026] using the query “Human orthorubulavirus 4” [porgn:__txid2560526], including the prototype strains AB543336 (hPIV4a), AB543337 (hPIV4b). A total of 656 sequences were initially retrieved. Only near-complete genome sequences with minimal ambiguous nucleotides, without large internal gaps and with available metadata were retained for analysis. After applying these criteria and removing incomplete or low-quality sequences, 170 strains (Supplementary Table S1), were included and aligned using MAFFT software version 7.

### 2.10. Nucleotide Identity Analysis

Pairwise nucleotide identity was calculated using the complete genome multiple sequence alignment of all hPIV4 strains included in this study. Pairwise percentage identities were computed using a custom Python script based on the Biopython v1.81, NumPy (Numerical Python) for numerical operations and Pandas (Python Data Analysis Library) for tabular data handling. For each sequence pair, the identity was determined by comparing each sequence pair position-by-position, counting matches over aligned, non-gap nucleotide positions. The resulting identity values were averaged to obtain within-group identities for Russian strains, identity of the Russian strains to the prototype strains and mean identity between hPIV4a and hPIV4b strains.

### 2.11. Data Availability Statement

The Genbank accession numbers for the nucleotide sequences determined in this study are PX353455–PX353462.

## 3. Results

### 3.1. Prevalence of hPIV

**Figure 1:**
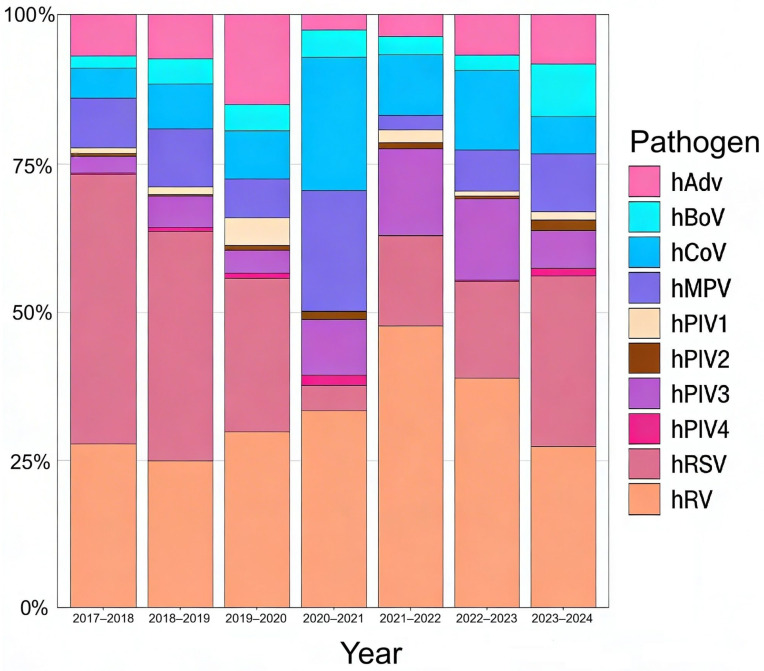
The percentage distribution of acute respiratory viral infections between 2017 and 2024. hAdV—human adenovirus, hBoV—human bocavirus, hCoV—human seasonal coronavirus, hMpV—human metapneumovirus, hPIV1—human parainfluenza virus type 1, hPIV2—human parainfluenza virus type 2, hPIV3—human parainfluenza virus type 3, hPIV4—human parainfluenza virus type 4, hRSV—human respiratory syncytial virus, hRV—human rhinovirus; Co-infections, although present, are not represented in this figure and are summarized in Supplementary Table S4. Influenza- and SARS-CoV-2 data were excluded from analysis to avoid skewing the proportional representation and visualization of the respiratory pathogens. A distribution including influenza viruses and SARS-CoV-2 is provided in Supplementary Figure S1 for epidemiological context.

During the study period from October 2017 to September 2024, a total of 19,213 respiratory samples were analyzed within the hospital-based surveillance program in Saint Petersburg. Human parainfluenza viruses were detected in 1508 samples (out of 12,355 samples positive for acute respiratory viral infections (ARVI), as determined by real-time PCR testing, which makes 12.2%) (Figure 1), including 71 cases of hPIV4. No simultaneous infections with multiple hPIV subtypes were observed, whereas co-infections with other respiratory viruses were relatively common (Supplementary Table S4). For downstream virological and genomic analysis, samples with Ct < 25 (*n* = 25) were selected for virus isolation, and further whole-genome amplification, and sequencing.

### 3.2. Virus Isolation on LLC-MK2 and MA-104 Cell Lines

**Figure 2:**
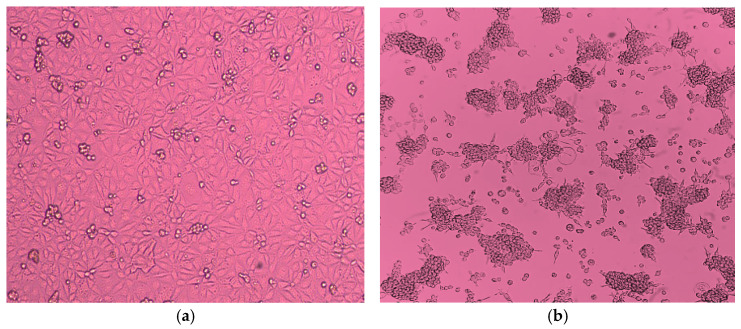
Microscopic images of LLC-MK2 cells infected with hPIV4; Scale bar = 100 µm: (**a**) left image shows negative viral isolation of hPIV4, no signs of CPE; (**b**) right image shows positive viral isolation of hPIV4 on LLC-MK2 cell culture, which manifested as enlarged rounded cells, cell-to-cell fusion, formation of syncytia, and cell lysis.

All hPIV4 samples with Ct values < 25 were subjected to virus isolation on LLC-MK2 and MA-104 cell lines. Three successive passages were performed for each sample. Eight samples out of twenty five were successfully isolated. The cytopathic effect was observed between 14 and 21 days after propagation (mean: 17 days), with more observable changes in LLC-MK2 cells. The CPE was characterized by the appearance of enlarged rounded cells, cell-to-cell fusion, formation of syncytia, and eventual cell lysis. As the subsequent passage progressed, the cytopathic effect became increasingly severe, leading to the complete lysis and detachment of the cell monolayer (Figure 2). The virus isolation was further examined through hemagglutination assay. All isolates tested negative in the hemagglutination assay performed with 0.5% suspensions of guinea pig, chicken, and human erythrocytes. The success of virus isolation was validated by real-time RT-PCR of the culture supernatant from the third passage, using commercial AmpliSens^®^ ORVI-screen-FL kits. While the clinical materials had cycle threshold (Ct) values range of 14–23, the amplified isolates showed markedly reduced Ct values (<10), confirming efficient viral propagation. hPIV4 isolates were subsequently preserved in the virus collection of Smorodintsev Research Institute of Influenza, (St. Petersburg, Russia). One strain hPIV4/Russia/SPE-RII-1446V1/2023 has been deposited in the State collection of viruses at the D.I. Ivanovsky Institute of Virology of the N.F. Gamaleya Research Center for Epidemiology and Microbiology, Ministry of Health of the Russian Federation, for use in future virological, molecular and epidemiological studies.

### 3.3. SMART-9N Whole-Genome Sequencing

**Figure 3:**
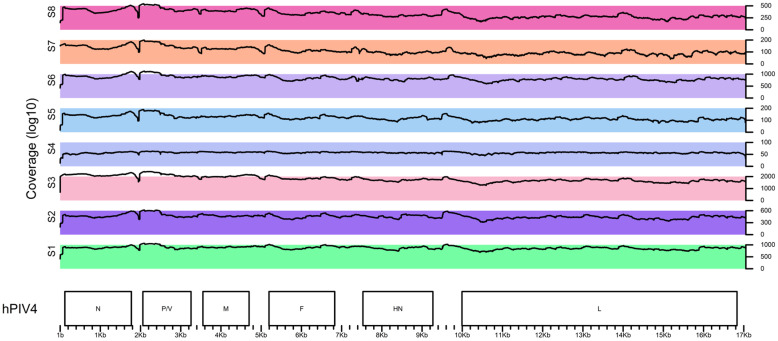
Coverage plot representing hPIV4 isolates’ genomes generated using the VizCoV script in the RStudio 2025.09.3+425 environment (https://github.com/LMV-NIC-St-Petersburg/VizCoV, accessed on 10 March 2026).

Complete whole-genome sequences were successfully recovered for all eight hPIV4 isolates using the SMART-9N metagenomic approach, followed by next-generation sequencing. For each isolate, full genome coverage was achieved, with no uncovered regions across the viral genomes (Figure 3). Coverage analysis demonstrated uniform read distribution across both structural and non-structural genes. The median coverage—ranging from 62× to 1306.5× across the analyzed isolates—was sufficient to establish high quality consensus genome sequences. More than 90% of genome positions were covered at ≥20× depth for all samples, and the proportion of genome positions with zero coverage was 0%. No systematic interruptions in coverage were detected at genome termini or within any coding regions, indicating that SMART-9N amplification provided consistent genome recovery. These results demonstrate that SMART-9N is a robust approach for generating complete hPIV4 genomes suitable for downstream phylogenetic genomic analysis.

### 3.4. Whole-Genome Phylogenetic Analysis

**Figure 4:**
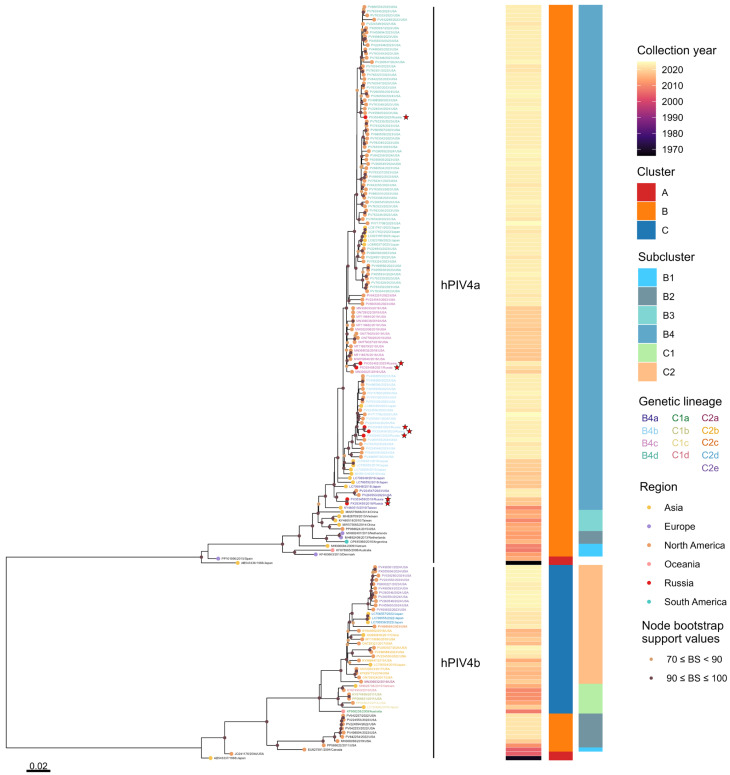
Maximum-likelihood phylogenetic tree based on the complete genome sequence of 178 hPIV4 strains. The tree was constructed using IQ-TREE v3.0.1. hPIV4 subtype classification is remarked with vertical lines. Clusters, subclusters and genetic lineages of each subtype are shown. Sequence identifiers are color-coded according to lineage assignment. Geographic regions are indicated by colored circles and the eight Russian isolates are highlighted by red circles and marked with stars. Accuracy of the tree was confirmed by applying 1000 bootstrap replicates, and values exceeding 70% are only denoted at the branches’ nodes. The scale bar indicates the number of nucleotide substitutions per site. Detailed metadata, including clusters, subclusters and genetic lineages assignments for all sequences, are provided in Supplementary Table S3.

A maximum-likelihood phylogenetic tree constructed from 178 complete hPIV4 genome sequences, including eight Russian isolates obtained in this study and 170 global genomes (Supplementary Tables S1–S3, provided as separate sheets within a single Excel file), identified two clearly separated subtypes, hPIV4a and hPIV4b (Figure 4). Subtype assignment based on whole-genome p-distance relative to the prototype strains hPIV4a M-25 (GenBank accession No. AB543336) and hPIV4b 68-333 (GenBank accession No. AB543337) identified 132 hPIV4a and 46 hPIV4b genomes. The mean nucleotide divergence between the two subtypes was 0.151, whereas the mean within-subtype diversity was 0.0149 and 0.0319 for hPIV4a and hPIV4b, respectively.

Within hPIV4a, two major clusters A and B were defined based on tree topology and p-distance, with genetic divergence exceeding 5.9% between them. Cluster A comprises the prototype strain M-25, isolated in Japan in 1966, together with a single Spanish strain, MBC050 (GenBank accession No. PP101996), exhibiting a mean within-cluster p-distance of 0.0198. Cluster B represents a genetically diverse group and is further subdivided into four subclusters B1, B2, B3 and B4. Subcluster B1 includes three strains originating from Denmark (HPIV4_DK(459), GenBank accession No. KF483663), Australia (HPIV-4a_QPID08-0015, GenBank accession No. KF878965) and Vietnam (HPIV4/Vietnam/112/2009, GenBank accession No. MH006684). Subcluster B2 consists of one Argentinian strain HPIV4a-HNRG (GenBank accession No. OP615960) and two Dutch strains t146a296_HPIV4 and t146a303_HPIV4 (GenBank accession No. MH892407 and MH892408), with a mean internal divergence of 0.0272. Subcluster B3 comprises five strains from Taiwan TW-00029-2010 (GenBank accession No. KY460518), Vietnam vzhpiv42 (GenBank accession No. MH828709), the United States HPIV4/USA/12N9/2015 (GenBank accession No. PP068624) and China BCH4237A/2014 and BCH4263A/2014 (GenBank accession No. MW575665 and MW575666). Subcluster B4 is the predominant group, encompassing 119 genomes from multiple continents. Mean genetic distances among the hPIV4a subclusters are 0.0320 (B1:B2), 0.0313 (B1:B3), 0.0382 (B1:B4), 0.0210 (B2:B3), 0.0276 (B2:B4), 0.0231 (B3:B4) (Table 1).

To further resolve the structure of the dominant hPIV4a subcluster B4, genetic lineages were defined using a 1.9% whole-genome p-distance cutoff, corresponding to the upper limit of the lineage-level divergence range (0.010–0.019) adopted from Mao et al. (2012) [38] and specified in the Methods section. Four multiple-sequence genetic lineages were identified: B4a (eight genomes), B4b (twenty-four genomes), B4c (nineteen genomes) and B4d (sixty-eight genomes). Mean intra-lineage diversity ranged from 0.0063 to 0.0125, while mean inter-lineage divergence ranged from 0.0104 to 0.0173 (Table 2), indicating the presence of distinct yet closely related lineages within the subcluster B4.

All eight Russian hPIV4 isolates clustered within hPIV4a, cluster B, subcluster B4, and were distributed among the four genetic lineages B4a, B4b, B4c and B4d. Two isolates hPIV4/Russia/SPE-RII-1011V2/2018 and hPIV4/Russia/SPE-RII-1011V1/2018 (GenBank accession No. PX353455 and PX353459) were assigned to lineage B4a, showing close genetic relatedness to strains previously detected in the United States, Japan and Taiwan. Three isolates hPIV4/Russia/SPE-RII-1446V1/2023, hPIV4/Russia/SPE-RII-2697V1/2023 and hPIV4/Russia/SPE-RII-1446V2/2023 (GenBank accession No. PX353456, PX353457 and PX353461) clustered within lineage B4b alongside strains from the United States, Japan and India. Two isolates hPIV4/Russia/SPE-RII-27084V1/2021 and hPIV4/Russia/SPE-RII-21726V2/2023 (GenBank accession No. PX353458 and PX353462) were classified within lineage B4c and grouped with strains from the United States. The remaining Russian isolate, hPIV4/Russia/SPE-RII-1280V2/2023 (GenBank accession No. PX353460) belonged to lineage B4d, close to strains from the United States and Japan. The mean whole-genome p-distance between the Russian strains and the hPIV4a prototype M-25 was 0.0647, while the mean divergence among the Russian strains was 0.0129.

The hPIV4b sequences segregated into three clusters A, B and C, with genetic divergence exceeding 4.6%. Cluster A comprises the prototype 68-333, isolated in Japan in 1968, and a closely related US strain 04-13 (GenBank accession No. JQ241176), with a mean internal divergence of 0.0154. Cluster B includes nine genomes from the United States and Canada, with a mean within-cluster p-distance of 0.0103, and is subdivided into two subclusters: subcluster B1 containing the single Canadian strain SKPIV4 (GenBank accession No. EU627591), and subcluster B2, comprising the remaining eight US strains. Cluster C encompasses thirty-five strains from Australia, the United States, Japan, China and Vietnam, exhibiting an intra-cluster diversity of 0.0186. Cluster C is further subdivided into two subclusters C1 and C2, each comprising multiple genetic lineages (four and five genetic lineages, respectively; Supplementary Table S3).

### 3.5. HN-Based Phylogenetic Analysis and Amino Acid Variations

**Figure 5:**
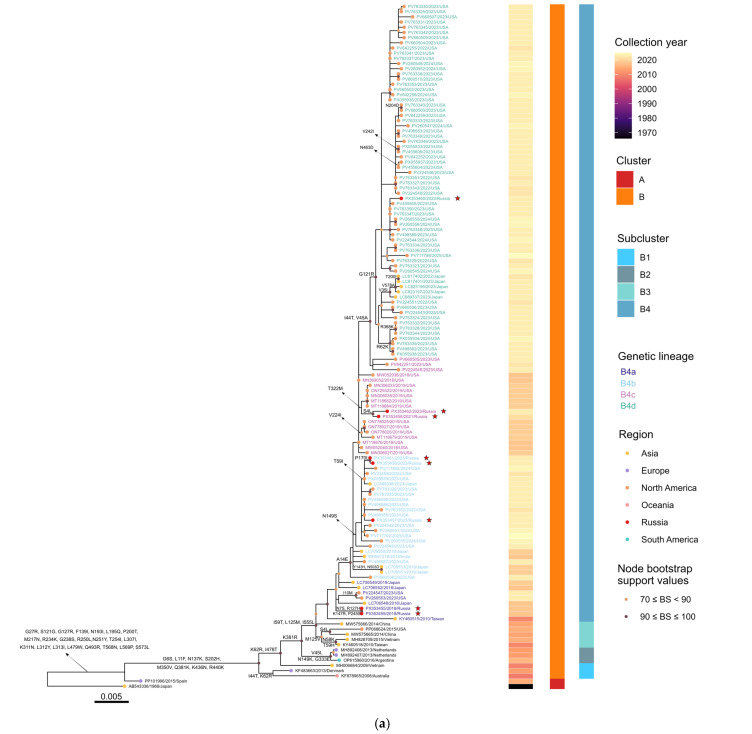

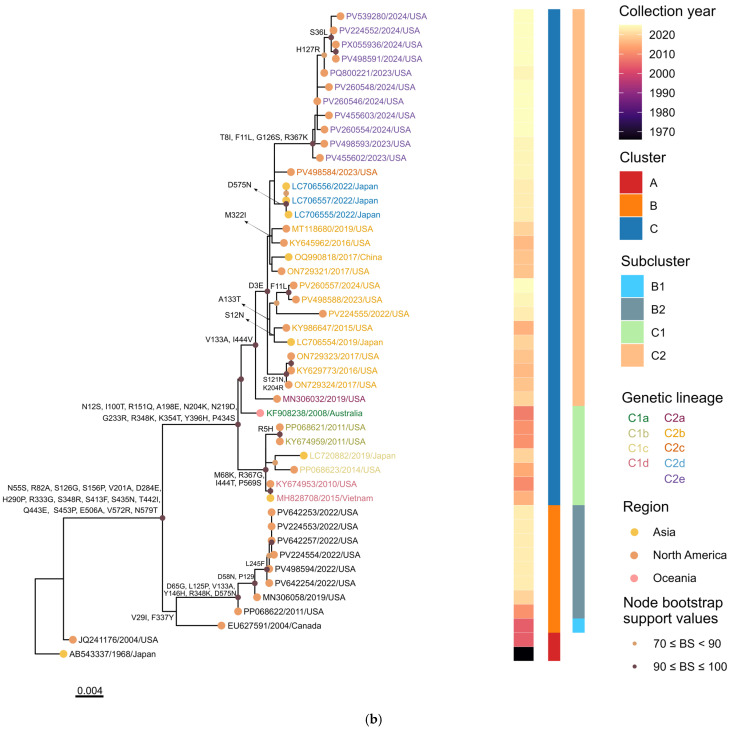
(**a**,**b**). Maximum-likelihood phylogenetic trees based on the complete HN gene sequence of 132 hPIV4a and 46 hPIV4b strains, respectively. The trees were constructed using the TreeSub tool (combining RAxML for phylogenetic inference and PAML for ancestral state reconstruction). Clusters, subclusters and genetic lineages of each subtype are shown. Sequence identifiers are color-coded according to lineage assignment. Geographic regions are indicated by colored circles and the eight Russian isolates are highlighted by red circles and marked with stars. Accuracy of the trees was confirmed by applying 1000 bootstrap replicates, and values exceeding 70% are only denoted at the branches’ nodes. The scale bars indicate the number of nucleotide substitutions per site.

Phylogenetic analysis based on the complete HN gene further resolved the evolutionary relationships within hPIV4 and was largely concordant with the topology inferred from whole-genome sequences (Figure 5a,b). Within each subtype, the HN-based trees were consistent with the subtype assignments established by whole-genome analysis and reproduced the major cluster and subcluster structure observed at the genome-wide level. Within hPIV4a, the HN tree reflected the same hierarchical organization as the genome-wide analysis, with two main clusters and multiple subclusters and genetic lineages. All Russian isolates grouped within the dominant hPIV4a B4 subcluster, consistent with their placement in the whole-genome phylogeny. Similarly, the HN-based phylogeny of hPIV4b was consistent with the whole-genome topology, resolving three main clusters with additional subcluster- and lineage-level diversification.

To investigate molecular features underlying this structure, amino acid substitutions in the HN protein were inferred along the phylogenetic branches using ancestral state reconstruction. Several amino acid changes were found to recur within specific clusters, subclusters and genetic lineages.

Within hPIV4a, the HN-based phylogeny revealed amino acid variation patterns that were largely consistent with the phylogenetic grouping. At the cluster level, cluster B was characterized by a recurrent set of substitutions, including G6S, L11F, N137K, S202H, M350V, Q381K, K436N, and R440K, which were present across most sequences in this cluster. Additional heterogeneity was observed at the subcluster level. Subcluster B1 predominantly exhibited substitutions I44T and K62R; however, one strain (MH006684) lacked these changes and instead shared substitutions (K62R, I478T) with more distantly related subclusters, reflecting ongoing diversification within this group. Subcluster B2 was associated with substitutions V45I, N149K, and G333E, whereas subcluster B3 shared I59T, L125M, and I555L across its members. In contrast, subcluster B4, which represents the dominant and most diverse group, did not display a single unifying amino acid pattern; instead, substitutions were distributed across multiple internal branches, consistent with extensive lineage-level diversification.

For hPIV4b, amino acid variation patterns were likewise structured according to phylogenetic grouping. Cluster B was associated with substitutions V29I and F337Y, while subcluster B2 additionally harbored D65G, L125P, V133A, Y146H, R348K, and D575N. Cluster C was characterized by a broader set of substitutions, including N12S, I100T, R151Q, A198E, N204K, N219D, G233R, R348K, K354T, Y396H, and P434S. Within cluster C, further subcluster-level patterns were evident: with the exception of the Australian strain KF908238, all strains in subcluster C1 shared M68K, R367G, I444T, and P569S, whereas subcluster C2 consistently exhibited V133A and I444V. Additional substitutions were observed within individual genetic lineages, reflecting continued genetic diversification within the subtype.

### 3.6. Potential N-Linked Glycosylation Sites in the HN Protein

Analysis of the HN protein of hPIV4a sequences revealed six predicted N-linked glycosylation motifs (score > 0.5), located at amino acid positions 279, 339, 347, 433, 502 and 530. These motifs were highly conserved across the majority of hPIV4a strains included in this study. All eight Russian strains possessed the full set of predicted glycosylation sites, with one notable exception: hPIV4/Russia/SPE-RII-27084V1/2021 (PX353458) lacked the glycosylation motif at position 279 due to the substitution T281I, which altered the canonical N-X-S/T motif and thereby disrupted the sequence pattern required for N-linked glycosylation. This substitution resulted in the loss of a predicted glycosylation motif that was otherwise conserved among the analyzed hPIV4a sequences.

In hPIV4b, five glycosylation motifs were identified at positions 279, 339, 347, 502 and 530. These motifs are present in nearly all hPIV4b strains analyzed. Notably, the 433 site observed in hPIV4a was absent in hPIV4b, indicating a potential subtype-associated difference in HN glycosylation pattern.

### 3.7. Pairwise Nucleotide Identity Analysis

Pairwise nucleotide identity analysis of the complete genome alignment showed that the average identity among the Russian hPIV4a strains 98.4%. In comparison to the hPIV4a prototype strain (M-25, AB543336), the Russian sequences exhibited nucleotide identities ranging from 93.4% to 94.0%. The closest relative of the Russian strains was a hPIV4a strain from Asia, Japan (LC706548), showing more than 97% identity. The mean nucleotide identity between hPIV4a and hPIV4b prototypes AB543336 and AB543337 was 87.7%.

## 4. Discussion

While most epidemiological and molecular studies have focused on hPIV 1, 2, and 3 types, hPIV4 has traditionally received limited scientific attention, largely due to its perceived low prevalence, frequent association with mild disease and historically poor recovery in cell culture [12,27,30]. Despite this, hPIV4 continues to be detected globally, which may be explained by its often mild or asymptomatic clinical presentation, potentially leading to underdiagnosis and sustained low-level transmission in the population. However, with the development of the routine multiplex PCR panels of respiratory virus detection in respiratory specimens, it has become occasionally more detectable [25,29,39] and an increasing number of studies suggest that its clinical and epidemiological significance may be greater than previously appreciated [26,27,28,29,30,31]. Nevertheless, comprehensive analyses of full-length hPIV4 genomes remain scarce, as the majority of published reports has concentrated on individual viral genes rather than whole-genome analysis.

In the last decade of the nineties, a series of studies conducted in Japan provided the first molecular characterization of hPIV4 by determining the nucleotide sequence of several individual genes, including NP [40], P [41], M [42], HN [43] and F genes [44] of both hPIV4a and hPIV4b subtypes. These early investigations laid the foundation for understanding the genomic organization of hPIV4, but were limited to partial genome genes. Subsequently, Lau et al. (2005) reported the first documented outbreak of hPIV4 infection in China and sequenced the complete phosphoprotein genes from the index cases [29]. Phylogenetic analysis of the deduced amino acid phosphoprotein sequences, performed in comparison with other members of the family Paramyxoviridae, demonstrated that the outbreak strains were more closely related to hPIV4a than to hPIV4b, providing early evidence of subtype-specific genetic divergence.

In 2006, Vachon et al. performed phylogenetic analysis based on nucleotide sequences of the two surface glycoproteins F and HN, from nine isolates and further highlighting the genetic heterogeneity of hPIV4 and supporting the distinction between hPIV4a and hPIV4b strains [12].

A major milestone was achieved in 2009, when Yea et al. reported the first complete genome sequence of hPIV4b, obtained from a clinical isolate (SKPIV4) collected at the Hospital for Sick Children in Toronto, Canada [45]. This was followed by the work of Komada et al. (2011), who completed the sequence analysis of both of hPIV4a and hPIV4b genomes by determining the L gene sequences and characterizing gene start, intergenic and gene end regions [46].

Further whole-genome-based investigations remained limited but included a comparative genomic analysis by Lednicky et al. (2012) of a full-length hPIV4b genome (isolate 04-13) [47]. Shortly thereafter, Abiko et al. (2013) described an Outbreak of hPIV4 infection among children with acute respiratory illnesses during the 2011–2012 winter season in Yamagata, Japan, performing phylogenetic analysis based on partial HN gene sequences [30]. Among the reported cases, seven were identified as hPIV4a and eleven as hPIV4b. In the same year, Alquezar-Planas et al. (2013) reported the complete genome sequence of a divergent hPIV4a from Denmark (HPIV4_DK(459)) using a viral metagenomic approach, showing that this strain clustered with the previously sequenced hPIV4a prototype (AB543336) [48]. In parallel, Bialasiewicz et al. (2014), characterized the full coding sequence of a novel hPIV4 variant from an adult with influenza-like illness, in Australia, demonstrating that the strain QLD-01 represented a divergent member of the hPIV4b subtype, most closely related to the SKPIV4 strain [49].

Despite these advances, most subsequent studies have continued to focus on individual genes—particularly P, F and HN—rather than complete genomes [15,19,50,51,52,53], leaving substantial gaps in our understanding of the global genomic diversity and evolutionary dynamics of hPIV4. In the present study, we addressed this limitation by applying a metagenomic whole-genome amplification approach based on the SMART-9N (Switching Mechanism at the 5′ end of RNA Template) technology [32,35]. This approach, which has not been previously applied to hPIV4, enables sequence-independent amplification of viral RNA without the need for prior primer design and allows reliable recovery of complete genomes with sufficient coverage for robust phylogenetic and comparative analysis. In contrast to amplicon-based approaches such as ARTIC, which rely on predefined primer sets and may result in uneven coverage or gaps in highly variable regions, the SMART-based method facilitates more uniform and complete genome recovery.

To define the phylogenetic structure of hPIV4, we adopted a genetic distance-based classification approach, which has been widely applied in the molecular characterization of hPIV3 [38,54,55,56]. Notably, Zhou et al. applied this criterion to the HN gene of hPIV4, using nucleotide divergence thresholds to define clusters, subclusters and genetic lineages within hPIV4a [51]. Building upon this established methodology, we applied a similar genetic distance-based approach to whole-genome sequences in the present study, which captures evolutionary signals from both structural and non-structural genes and therefore provides greater phylogenetic resolution than single-gene analysis while remaining comparable to previously established classification frameworks.

Using this whole-genome p-distance-based framework, our analysis provides, to the best of our knowledge, the first comprehensive classification of hPIV4 based on complete genome sequences. Whole-genome phylogenetic analysis of 178 hPIV4 strains confirmed the clear separation of hPIV4 into the two recognized subtypes, hPIV4a and hPIV4b, with substantial nucleotide divergence between them, consistent with their long-established antigenic and genetic distinction. Within hPIV4a, two deeply separated clusters were resolved (A and B), corresponding to a prototype-associated cluster and a genetically diverse cluster, encompassing the majority of globally circulating strains. The latter exhibited further hierarchical structuring into multiple subclusters (B1–B4) and genetic lineages (B4a–B4d), highlighting the substantial genomic diversity present within contemporary hPIV4a sequences.

Notably, all Russian isolates obtained in this study were positioned within the dominant hPIV4a cluster B, subcluster B4, and further distributed across several genetic lineages, indicating co-circulation of multiple lineages rather than the emergence of geographically restricted variants. Their genetic distances relative to the prototype strain support the interpretation that hPIV4 viruses circulating in Russia represent modern descendants of widely disseminated lineages. Identity analysis further confirmed this conclusion, demonstrating that the Russian genomes shared approximately 98.4% identity with one another, 93.4–94.0% identity with the prototype hPIV4a strain.

A similar hierarchical structure was observed within hPIV4b, which demonstrates three distinct clusters (A, B and C) and subclusters with divergence values comparable to those reported in earlier phylogenetic analyses reinforcing the robustness of the whole-genome p-distance approach.

Importantly, phylogenetic analysis based on the complete HN gene was largely concordant with the whole-genome topology for both subtypes. The HN-based trees reproduced the major cluster and subcluster structure observed at the genome-wide level, supporting the stability of the inferred evolutionary relationships. This concordance indicates that the genome-wide classification is not driven by a single gene but reflects broader evolutionary signals across the viral genome. In addition, analysis of amino acid substitutions in the HN protein revealed patterns that were broadly consistent with phylogenetic grouping. While no single substitution uniquely defined all clusters or lineages, recurrent amino acid changes were observed within specific clusters, subclusters, and genetic lineages, reflecting progressive diversification within established phylogenetic frameworks.

Molecular characterization of the HN protein revealed conserved subtype-specific differences in predicted N-linked glycosylation motifs. hPIV4a strains possess six predicted glycosylation sites, including a site at position 433, which is not present in hPIV4b. This motif-level distinction may contribute to structural or antigenic differences between the subtypes. All eight Russian strains displayed the complete set of predicted hPIV4a glycosylation sites except for one strain, which lacks the motif at the position 279 due to disruption of the N-X-S/T pattern. Glycosylation plays a key role in shielding viral surface proteins from neutralizing antibodies and modulating receptor-binding affinity; thus, loss of a conserved glycan warrants further investigation [50,57]. Recent structural analyses of the hPIV4 HN protein have identified conserved catalytic residues involved in neuraminidase activity (R175, D199, E402, R417, R507, Y535, and E556), all located within the globular head domain [53]. Importantly, none of the predicted glycosylation sites identified in the present study overlaps with these catalytic residues. Therefore, the subtype-specific glycosylation differences observed here, including the absence of the 433 site in hPIV4b and the loss of the 279 motif in one Russian isolate, are unlikely to directly affect the enzymatic active site. Instead, these glycan variations may influence local surface conformation or accessibility within the head domain rather than the core catalytic machinery.

The continued global circulation of hPIV4 despite its relatively low detection frequency may be explained by several factors. Infections are often mild or asymptomatic, which likely contributes to underdiagnosis and sustained community transmission. In addition, the co-circulation of multiple genetic lineages observed in this study supports ongoing viral persistence rather than localized or sporadic introduction events. Together, these observations suggest that hPIV4 may circulate at low detectable levels while maintaining a stable global presence.

## 5. Conclusions

In conclusion, this study provides the first complete genome sequences of hPIV4 viruses circulating in Russia and places them within the context of global hPIV4 diversity. All isolates belong to the contemporary hPIV4a subcluster B4, showing close genetic relatedness to globally circulating strains and supporting the co-circulation of diverse lineages within a single geographic region. Phylogenetic analysis, glycosylation patterns and HN-based amino acid variation analysis collectively highlight the ongoing evolution and genetic diversity of hPIV4. Importantly, this work represents the first application of SMART-based whole-genome amplification to hPIV4, demonstrating that this approach is a robust and efficient strategy for recovering complete viral genomes from cell culture isolates. The successful application of this method underscores its value for future genomic surveillance of hPIV4 and other respiratory viruses. These findings expand the current knowledge of hPIV4 genetic diversity, emphasize the importance of genome-level surveillance and provide a framework for future molecular epidemiological studies aimed for identifying circulating strains, tracking viral evolution and monitoring the possible emergence of variants with altered biological or antigenic properties.

## 6. Limitations

This study has several limitations. First, the number of analyzed Russian isolates is relatively small (*n* = 8), which may not fully capture the diversity of hPIV4 circulating in the region. Second, the set of publicly available genomes used for comparative analysis, although comprehensive, does not necessarily reflect the full global diversity of hPIV4, as genomic data remain unevenly distributed across geographic regions. Finally, hPIV4 remains comparatively understudied relative to other respiratory viruses, which limits the availability of reference data and may constrain broader evolutionary and epidemiological interpretations.

## Figures and Tables

**Table 1:** Estimates of genetic distances over sequence pairs among the subclusters of hPIV4a cluster B.

	B1	B2	B3	B4
B1				
B2	0.0320			
B3	0.0313	0.0210		
B4	0.0382	0.0276	0.0231	

**Table 2:** Estimates of genetic distances over sequence pairs among the four genetic lineages of subcluster B4 of hPIV4a.

	B4a	B4b	B4c	B4d
B4a				
B4b	0.0162			
B4c	0.0139	0.0112		
B4d	0.0173	0.0143	0.0104	

## Data Availability

Sequences of all used genomes were deposited to Genbank (Accession Numbers: PX353455–PX353462.

## Supplementary Materials

The following supporting information can be downloaded at: https://www.mdpi.com/article/10.3390/v18050497/s1, Table S1: 170 complete genome sequences of hPIV4 strains used in the current study; Table S2: 8 Russian complete genome sequences of hPIV4 isolates obtained in the current study; Table S3: 178 Genbank and Russian complete genome sequences of hPIV4 strains used in Figure 4. Table S4: Co-infections of human parainfluenza viruses (hPIV) with influenza viruses and SARS-CoV-2 in respiratory samples (2017–2024). Figure S1: The percentage distribution of acute respiratory viral infections-distribution, including influenza viruses and SARS-CoV-2, between 2017 and 2024.
