# Supplementary figures and images for: Whole-Genome Phylogenetic Characterization of Human Parainfluenza Virus Type 4 Circulating in St. Petersburg, Russia
Source: Viruses. 2026 Apr 24;18(5):497. doi: 10.3390/v18050497 (PMC13211376; doi:10.3390/v18050497)

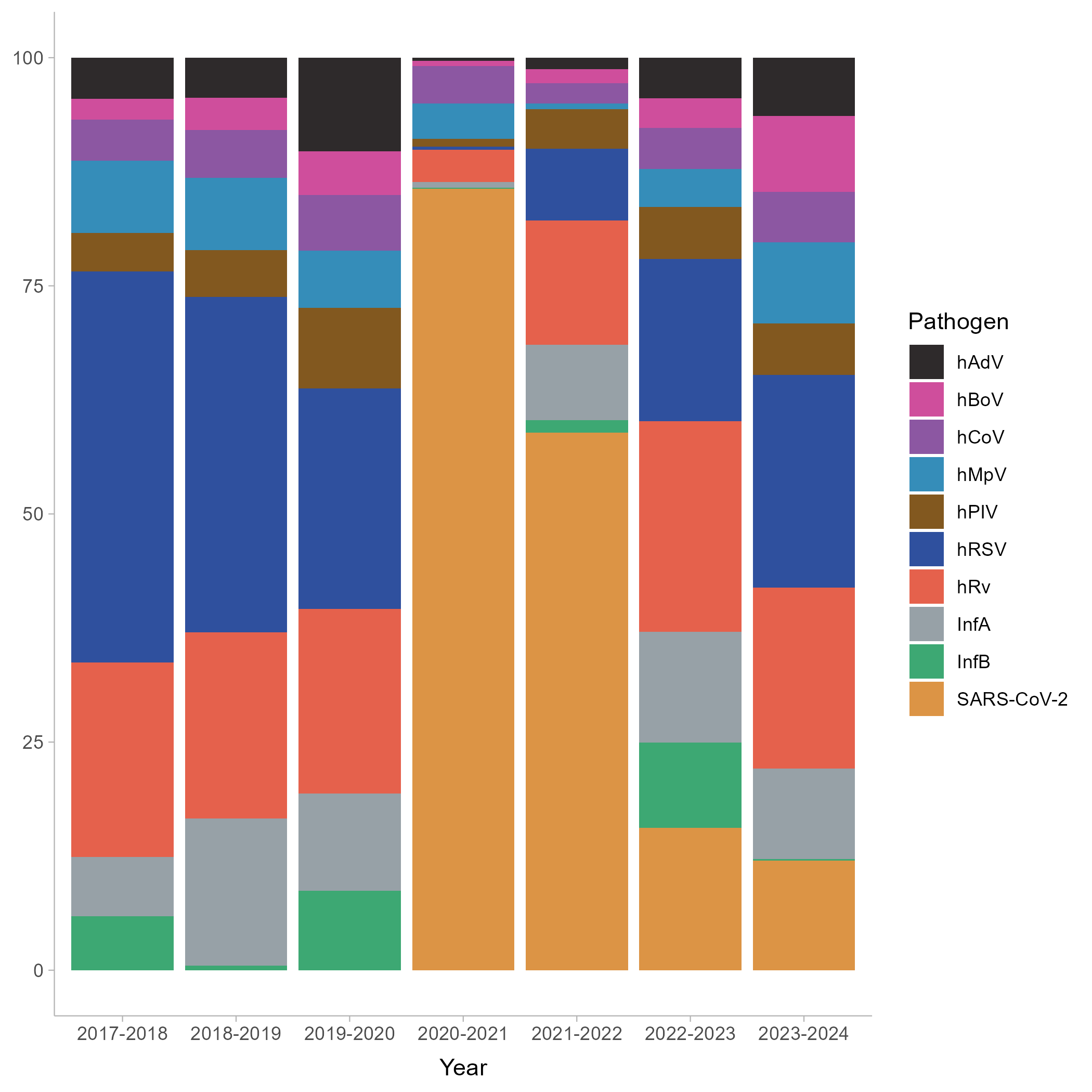

Supplement: Supplementary file 1 [file viruses-18-00497-s001.zip › Supplementary figure S1.png]
